# Strengthening healthcare providers’ capacity for safe abortion and post-abortion care services in humanitarian settings: lessons learned from the clinical outreach refresher training model (S-CORT) in Uganda, Nigeria, and the Democratic Republic of Congo

**DOI:** 10.1186/s13031-021-00344-x

**Published:** 2021-04-06

**Authors:** Nguyen Toan Tran, Alison Greer, Talemoh Dah, Bibiche Malilo, Bergson Kakule, Thérèse Faila Morisho, Douglass Kambale Asifiwe, Happiness Musa, Japheth Simon, Janet Meyers, Elizabeth Noznesky, Sarah Neusy, Burim Vranovci, Bill Powell

**Affiliations:** 1 Australian Centre for Public and Population Health Research, Faculty of Health, University of Technology Sydney, PO Box 123, Sydney, NSW 2007 Australia; 2 Faculty of Medicine, University of Geneva, Rue Michel-Servet 1, 1206 Genève, Switzerland; 3 Training Partnership Initiative of the Inter-Agency Working Group on Reproductive Health in Crises, Women’s Refugee Commission, 15 West 37th Street, New York, NY 10018 USA; 4 Federal Medical Centre, Keffi, Nasarawa State PMB 1004 Nigeria; 5 Save the Children International DRC, 16 Avenue Avenue des Ecoles, Quartier les Volcans, Commune de Goma, North Kivu, Democratic Republic of the Congo; 6 CARE International DRC, Kinshasa, Democratic Republic of the Congo; 7 CARE International Nigeria, 289 Amolai Road, GRA, Maiduguri, Nigeria; 8 Save the Children, 899 N Capitol Street, NE, Washington, DC 20002 USA; 9 CARE USA, 151 Ellis Street NE, Atlanta, GA 30303 USA; 10 Doctors of The World/Médecins du Monde, France Headquarters, 62 rue Marcadet, 75018 Paris, France; 11 Ipas, P.O. Box 9990, Chapel Hill, NC 27515 USA

**Keywords:** Safe abortion care, Post-abortion care, Capacity building, Refresher training, Human resources for health, Humanitarian settings, Sexual and reproductive health and rights

## Abstract

**Background:**

Fragile and crisis-affected countries account for most maternal deaths worldwide, with unsafe abortion being one of its leading causes. This case study aims to describe the Clinical Outreach Refresher Training strategy for sexual and reproductive health (S-CORT) designed to update health providers’ competencies on uterine evacuation using both medications and manual vacuum aspiration. The paper also explores stakeholders’ experiences, recommendations for improvement, and lessons learned.

**Methods:**

Using mixed methods, we evaluated three training workshops that piloted the uterine evacuation module in 2019 in humanitarian contexts of Uganda, Nigeria, and the Democratic Republic of Congo.

**Results:**

Results from the workshops converged to suggest that the module contributed to increasing participants’ theoretical knowledge and possibly technical and counseling skills. Equally noteworthy were their confidence building and positive attitudinal changes promoting a rights-based, fearless, non-judgmental, and non-discriminatory approach toward clients. Participants valued the hands-on, humanistic, and competency-based training methodology, although most regretted the short training duration and lack of practice on real clients. Recommendations to improve the capacity development continuum of uterine evacuation included recruiting the appropriate health cadres for the training; sharing printed pre-reading materials to all participants; sustaining the availability of medication and supplies to offer services to clients after the training; and helping staff through supportive supervision visits to accelerate skills transfer from training to clinic settings.

**Conclusions:**

When the lack of skilled human resources is a barrier to lifesaving uterine evacuation services in humanitarian settings, the S-CORT strategy could offer a rapid hands-on refresher training opportunity for service providers needing an update in knowledge and skills. Such a capacity-building approach could be useful in humanitarian and fragile settings as well as in development settings with limited resources as part of an overall effort to strengthen other building blocks of the health system.

## Background

Approximately two-thirds of maternal deaths worldwide occur in countries affected by fragility and crises [1]. Unsafe abortion is one of the leading causes of maternal mortality and morbidity, with 5–13% of attributed maternal deaths worldwide [2] and South Asia and sub-Saharan Africa accounting for an overwhelming majority of these deaths [3]. Therefore, emergency care for women with abortion complications (post-abortion care) and safe termination of pregnancy (safe abortion care) are lifesaving [4].

As such, the Minimal Initial Service Package (MISP) for Sexual and Reproductive Health (SRH) in humanitarian settings—an international standard in humanitarian response to be delivered from the onset of a crisis [5]—has a four-prong integration of these services into its objectives [6]. First, under the MISP objective on sexual violence, *pregnancy testing, pregnancy options information, and safe abortion care/referral for safe abortion care, to the full extent of the law* are components of the clinical care for survivors [6]. Second, post-abortion care is a signal function of emergency obstetric care and falls under the MISP objective to prevent excess maternal morbidity and mortality [6]. Third, ensuring that safe abortion care is available in health centers and hospitals to the full extent of the law is a standalone priority of the MISP [6]. Lastly, voluntary contraception, which is reflected in the MISP objective on preventing unintended pregnancy, is a key component of post-abortion and safe abortion care services [6].

Nonetheless, abortion-related knowledge (including of national laws), technical skills, and services are notably lacking in most crisis settings as illustrated by various evaluations and reviews, which found inconsistent to non-existent service delivery of contraception (in particular long-acting methods) and safe abortion care in crisis settings [7, 8] and called for special attention on this underserved issue [9]. Likewise, these services were under-represented in humanitarian appeals and funding allocations [10], and the topic received little attention in terms of health intervention research in humanitarian crises [11].

Safe abortion services may be perceived as too complicated to implement [12] or unrealistic to offer openly in humanitarian contexts [13]. In terms of required clinical competency, mid-level providers can safely perform uterine evacuation using manual vacuum aspiration (MVA), medications, or both, after appropriate training [14]. To help ensure there is clinical staff available with the knowledge and skills to provide these services in crisis-affected and fragile settings, Ipas and the Training Partnership Initiative of the Inter-Agency Working Group for SRH in Crisis-Settings collaborated to develop a refresher training course on uterine evacuation covering both MVA and medication approaches. The course adopted an established capacity-building strategy known as the S-CORT (SRH Clinical Outreach Refresher Training) [15]. The model is not meant to build the capacity of people who have not been previously trained on MVA. Instead, it aims to reach out to frontline health providers working in humanitarian contexts, such as nurses and midwives, and refresh their knowledge and skills on lifesaving SRH skills, which they previously learned but may not have kept up to date. Such training courses last two to 3 days depending on the topic and usually do not include a clinical practicum. The uterine evacuation module was designed to be used either as a stand-alone one-day training focusing on the medications approach or as a two-day training combining both medications and MVA. In contrast to other S-CORT modules, the training on medication-based uterine evacuation is appropriate for either new learners or as a refresher course because it is a knowledge-based clinical service that does not require further hands-on clinical skills. This paper is a case study describing the design and contents of the S-CORT on uterine evacuation, lessons learned from its implementation, and stakeholders’ experience in humanitarian contexts in Uganda, Nigeria, and the Democratic Republic of Congo (DRC), as well as recommendations for improvement.

### Settings

In 2019, Uganda hosted close to 1.4 million refugees, with a majority from South Sudan, followed by DRC and Burundi [16]. Although the Ugandan Constitution declares that *no person has the right to terminate the life of an unborn child except as may be authorized by law* (Penal Code of 1950, Section 141)*,* its 2012 SRH policy clarified the exceptions to the rules allowing induced abortion under specific circumstances, including rape and incest as well as severe maternal and fetal conditions [17]. However, these exceptions have not been made explicit and consistent within the law and across policies, resulting in a lack of knowledge, understanding, and coherent application of the law, and concern among clinical providers about being penalized for providing abortion services, which impacts their availability [18]. Approximately 375 women die from pregnancy-related causes out of every 100,000 live births nationwide [1]. Unsafe abortion is seen as a major contributor to maternal mortality and there is a high demand for but insufficient access to safe abortion services. A study estimated that, in 2013, 52% of pregnancies were unplanned, 314,304 induced abortions were performed, and 128,682 women were treated for abortion-related complications in health facilities [19].

The protracted armed conflict in Northeast Nigeria resulted in around 1.8 million internally displaced people in 2019 [20]. The country’s restrictive laws, which differ in Northern and Southern Nigeria, permit induced abortion only to save a woman’s life [21]. Due to their criminalization, the majority of abortions occur in unsafe conditions, as illustrated by the estimated 1.25 million women nationwide who had an induced abortion in 2012 with the highest number in Northeast Nigeria [22]. Among the 1.25 million women, 212,000 received treatment for complications of unsafe abortions, while 285,000 experienced serious health consequences but did not receive treatment. The national maternal mortality ratio in 2017 was 917 per 100,000 live births [1].

In 2019, 12.8 million people needed humanitarian assistance in DRC, with the eastern region particularly affected by decades of armed conflict, political unrest, and fragility compounded in recent years by Ebola Virus Disease outbreaks [23]. Before March 2018, induced abortion was not legal under any circumstance in the DRC. However, an article in the code of medical ethics allowed doctors to perform the service to save a woman’s life [24]. In 2018, the DRC endorsed the African Charter on Human and People’s Rights on the Rights of Women in Africa (Maputo Protocol), which allowed women to legally access abortion under a broader range of conditions—including in cases of sexual assault, rape, or incest [25]. There is no published report on abortion incidence except for Kinshasa, where an estimated 37,865 women obtained treatment for induced abortion complications in health facilities in 2016 [26]. The maternal mortality ratio reported in 2017 was 473 per 100,000 live births [1].

Despite differences among the three countries regarding their abortion laws, all allowed access to postabortion care (emergency care for women with abortion complications), reflecting its legality globally [27]—even countries with highly restrictive abortion laws recognize postabortion care as a critical component of essential emergency obstetric care.

## Methods

### Intervention

The S-CORT approach is designed for individual or group-based training with a focus on participatory learning and skills practice. The training package comprises a facilitator’s guide (see https://iawg.net/resources/uterine-evacuation-in-crisis-settings-using-mva-refresher-training ) and slide sets with seven units for the trainer and handouts, checklists, and job aids for participants. The agenda is modular to accommodate a stand-alone medication or MVA training or a combination of both. The module on uterine evacuation using medication covers post-abortion care and safe abortion care (with combined mifepristone and misoprostol or misoprostol-only regimens). Training methodologies include slide-supported interactive presentations, group discussions, and questions and answers, case studies, small group work, role-plays, videos, and demonstration and hands-on skills practice with anatomical models along with checklists to guide practice. The training module contents drew from the latest available resource materials, including guidance from the World Health Organization (WHO) and Ipas *Woman-Centered, Comprehensive Abortion Care: Trainer’s Manual,* and were adapted for humanitarian contexts [4, 28].

### Selection of implementing partners

In 2019, Ipas and the Training Partnership Initiative of the Inter-Agency Working Group for SRH in Crisis-Settings partnered with three implementing organizations solicited through an open application process to pilot the module. Criteria for implementing organizations included having safe abortion care or post-abortion care, or both, within the organization’s workplan; prior experience organizing trainings in crisis-affected settings; commitment to supporting capacity development efforts for national and international SRH providers; and capacity to undertake an evaluation of the module and training workshop.

**Fig. 1:**
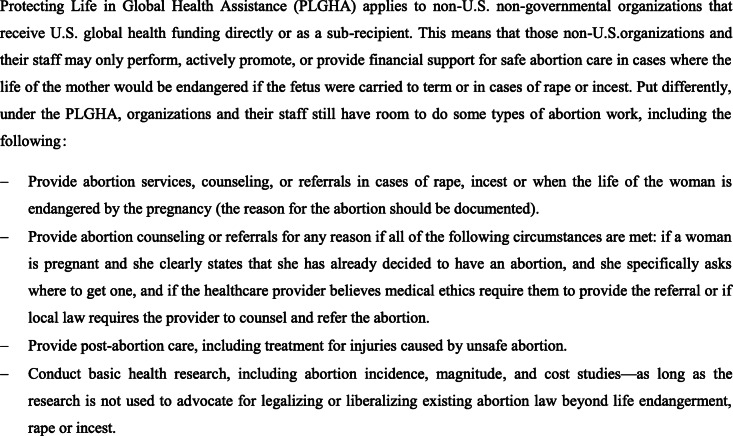
Adapting abortion and post-abortion care programs to the U.S. policy on Protecting Life in Global Health Assistance [29]

Pilots were held in Yumbe, Uganda, in April 2019 with Médecins du Monde France and in Maiduguri, Nigeria, in July 2019 with CARE Nigeria. Both trainings were conducted in English. For the third pilot held in Goma, DRC, in August 2019, the training materials were edited for compliance with the U.S. policy on Protecting Life in Global Health Assistance (PLGHA) (see Fig. 1). The materials were then translated into French, which was the language used during the training. This pilot was a collaboration with CARE DRC and Save the Children DRC. In each setting, an Ipas trainer from the national or international office was the lead or co-facilitator. These training courses were attended by doctors, nurses, midwives, and other mid-level providers. While many reported some prior exposure to or experience with MVA, only a few did so for uterine evacuation using medication.

Partners were provided the option to pilot the uterine evacuation module with medications alone or in combination with MVA. All selected the combined training. A values clarification and attitude transformation (VCAT) session is strongly recommended in advance of the uterine evacuation with medications and MVA trainings [30]. As such, a one-day VCAT workshop preceded the clinical training in Nigeria. In DRC, a VCAT exercise was integrated into the agenda of the first day. In Uganda, all participants had previously participated in a VCAT workshop.

### Evaluation objectives

The evaluation had two primary objectives: first, to evaluate the training materials themselves, including the clinical outreach training model, which would help inform the finalization of the design and contents of the training package, and, second, to evaluate the implementation of the training, which will strengthen the guidance around organizing such trainings.

The evaluation of the module adopted a mixed-method approach, which included a self-filled pre-test and post-test questionnaire, a competency checklist, a self-filled end-of-training evaluation, and qualitative interviews with participants.

### Pre-test and post-test

The questionnaire comprised 24 multiple-choice questions in English or French and was administered at the beginning and end of the training workshop. The questions aimed at assessing the level of knowledge related to the continuum of care for uterine evacuation from counseling to contraception. They stemmed from a pool of questions that Ipas had pilot-tested and utilized in numerous training workshops worldwide. Mean scores were computed.

### Competency checklist

Clinical competency was assessed at the beginning and at the end of the workshop using checklists comprising 63 systematic steps for medication uterine evacuation and 78 for manual vacuum aspiration. Anatomical models were used for MVA and role-plays for medication uterine evacuation. Due to their limited number, facilitators could not observe each participant and complete the checklist accordingly. This was done by a fellow participant under the supervision of a facilitation team member. Mean scores were computed. However, data from the competency checklists were incomplete and therefore unreliable for analysis.

### Qualitative data

At the end of the workshop, an independent evaluation staff from the main implementing partner conducted focus group discussions with participants. The evaluation teams used convenience sampling for participant selection by inviting trainees to participate and by ensuring equitable representation by gender and professional profiles. The focus group discussions were audiotaped after obtaining agreement from participants. Research assistants transcribed the audio recordings into English or French with accuracy checks done by comparing transcripts with audio files. Focus group discussions gathered male and female trainees, and, therefore, the transcriptions did not report the distinction between genders. Written comments from end-of-workshop evaluations by participants and feedback from trainers provided additional sources of qualitative data. Thematic analysis was performed by qualitative analysts fluent in both French and English using QSR NVivo 12 software, a qualitative research management tool. A basic codebook, which describes all the codes used for analysis, was pre-established based on the discussion guide and used to code data. The codebook was enriched with new codes as they emerged during the coding process.

### Ethics and informed consent

The pre- and post-workshop surveys and qualitative tools were part of planned program monitoring, which was not designed to develop and contribute to generalizable knowledge and, therefore, did not constitute research and require ethical approval [31]— this was confirmed by our submission to the Western Institutional Review Board (No. 2633824–44,635,729). The evaluation was deemed to pose no risk, and there was no requirement for workshop participants to complete the evaluation as a condition of workshop attendance and no incentive in participating in the evaluation. Therefore, there was no need for informed consent. Evaluators informed participants that their participation in the evaluation was voluntary, all their feedback would be anonymized, and its management and analysis handled confidentially. No patient participated in the evaluation.

## Results

Between 15 April and 31 August 2019, implementing partners ran a three-day or four-day pilot training workshop in each of the three participating countries (Table 1, Part 1). The nature of all three contexts was humanitarian or fragile. The number of participants ranged from 21 to 30 per workshop with a total of 72 people (35 women and 37 men). They were nurses, midwives, physicians, medical coordinators, and programmatic staff affiliated with the partnering organization. In Nigeria and the DRC, members of the Ministry of Health participated in the training. In Nigeria, there were ten community health extension workers and one radiologist—this was contrary to the recommended criteria for participants and part of the lessons learnt (see Discussion).

**Table 1:** Summary of key workshop characteristics (Part 1), applied evaluation tools, and results (Part 2) in Uganda, Nigeria, and DR Congo

	Uganda	Nigeria	DR Congo
**Part 1**			
Partners	Ipas, Médecins du Monde	Ipas, CARE	Save the Children, CARE, Ipas
Settings	Bidibidi refugee settlement, Yumbe District, West Nile region	Maiduguri, Borno State	Goma, North Kivu
Dates	April 2019	July 2019	August 2019
Number of participants	21 (9 women, 12 men)	21 (18 women, 3 men)	30 (8 women, 22 men)
Professions/ functions	Physicians, midwives, nurses, clinical officers, medical coordinators	Physician, midwives, nurses, community health extension workers (*n* = 10), radiologist (*n* = 1)	Clinical and programmatic staff from CARE and Save the Children
Duration	3 days - 1 day on medication uterine evacuation - 1 day on MVA uterine evacuation - 1 day for the validation of clinical competencies	4 days - 1 day on values clarification and attitude transformation - 1 day on medication uterine evacuation - 1 day on MVA - 1 day for the validation of clinical competencies	4 days - 1 day on values clarification and attitude transformation - 1 day on MA - 1 day on MVA - 1 day for the validation of clinical competencies
**Part 2**			
Pre−/post-test knowledge scores	- n with complete data = 18 - pre-test: 84% - post-test: 89%	- n with complete data = 20 - pre: 45% - post: 52%	- n with complete data = 27 - pre: 56% - post: 76%
Pre−/post-training competency checklist	In-training use but data not collected	In-training use but incomplete and unreliable data	In-training use but incomplete and unreliable data
Qualitative interview	1 FGD (3 women, 3 men) 1 IDI with co-trainer	2 FGD (corrupt audio files)	3 FGD with a total of 19 participants (5 women, 14 men)

The core curriculum comprised a day on manual vacuum aspiration and another one addressing medication-based uterine evacuation. On the basis of participants’ needs and available resources, facilitators added a first day dedicated to abortion values clarification and attitude transformation (Nigeria, DRC) and all three workshops included a day of validation of clinical competencies with real clients or through role-plays using humanistic models if no planned clients showed up. In all three countries, facilitators included a discussion on ways to integrate uterine evacuation into health facilities in humanitarian settings, which is part of the monitoring and evaluation chapter of the module.

In all three countries, results for the knowledge pre-test and post-test were available. As mentioned under Methods, data from the competency checklists were incomplete and therefore unreliable for analysis (Table 1, Part 2). In DRC, an evaluation officer conducted three focus group discussions with a total of 5 women and 14 men. In Uganda, due to limited resources, one of the facilitators had to conduct just one focus group discussion with 3 women and 3 men and another facilitator provided written feedback on the use of the facilitator’s guide. In Nigeria, there were two focus group discussions. However, the recording and audio files, which contained the details about the number of participants and their gender, were corrupt and therefore not usable.

### Pre-test and post-test

The average scores of participants rose significantly in all three countries but from different baselines and with different percentage point increase. In the DRC, the score increased from 56 to 76%; in Uganda, from 84 to 89% with the best improvement at + 25 percentage point; and in Nigeria, from 45 to 52% with the best improvement at 25 percentage point but a third of participants scored worse after than before. A participant in Uganda reported that the pre-test should be “less bulky, comprehensive, and cumbersome,” an impression echoed by participants from the other settings.

### Qualitative results

#### Confidence, skills building, and relevance

Participants from all three countries reported that the workshop enhanced their competencies, strengthened their confidence by overcoming fear to deliver uterine evacuation information and services, and eventually transformed their attitudes in relation to uterine evacuation.

*Before, I even feared to talk about it because I could not even defend my thoughts. I really feared when somebody came to me and talked about abortion: tell me more about it, what is the service? I really did fear because I lacked the evidence, and I didn’t know what I was doing…I used to fear the complications. But I have also learned about how to manage complications and even how they can come about during the process. I know how to help with some of the complications that may come about, how it can also be avoided during the process. –* Participant from Uganda.

When asked about what they would do differently as a consequence of the workshop, participants listed improving counseling, respecting all clients, and specific clinical procedures, including the administration of paracervical blocks or medication for pain control, as illustrated hereafter:

*What I would do differently? A paracervical block before doing manual vacuum aspiration, pain management using ibuprofen, and know how to administer mife [mifepristone] in combination with miso [misoprostol] or give miso alone.* – Participant from the DRC

*I will change my attitude. I will do follow up. I will do good counseling. I will have self-confidence, respect for all clients and provide quality care to all clients irrespective of age, religion, and marital status. –* Participant from Nigeria

The previous quote came from the Nigeria workshop and suggests that the S-CORT curriculum could influence attitudinal changes related to the quality of care and non-discrimination even without a dedicated day on values clarification and attitude transformation. In addition, the need for non-discrimination was repeatedly underscored as well as freedom from shame, as exemplified hereafter:

*This training has helped us not to discriminate anyone who has come for the service. So, you cannot discriminate this one who is young or this one who is old so you cannot do the procedure. It has helped us to do abortions to any client who really wants the service…I feared talking about abortion but now I’m okay because sometimes I see people dying, but I think helping these people about abortion is better than leaving them dying. And right now, I have come to really believe that with the knowledge I got, with the medical method and the evacuation, I can really help a lot of people in crisis and also, I will not feel so ashamed to talk with them, to counsel them so that I will not lose them.* – Participant from Uganda

Participants reported the need to have a more concrete discussion—and examples—on how to improve the integration of their uterine evacuation skills into their healthcare services, as most providers found the training workshops relevant to their job:

*This knowledge is very relevant to my profession being a comprehensive nurse. I have to know everything. Basic things in the medical profession so that I am able to handle any case. I cannot say this is a maternity case or this is a gyne[cological] case that has to be handled by midwives or doctors or something of that kind. So, I feel that this knowledge is very relevant to me so that it will help me to manage any case which presents to me.* – Participant from Uganda

#### Counseling, human rights, and the law

Participants seemed adamant about the effect of the training on the way they would do counseling, reporting that their counseling would be underpinned by human rights principles, such as client autonomy and choice. In addition, the workshop appeared to have helped clarify the country’s legal framework for service providers, paving the way for fearless counseling and service provision.

*I will do this service better since the grey areas I had were lifted with this training, since safe medical abortion is already allowed by the law of the country since it is a need felt in the population despite the ignorance of some*. – Participant from the DRC

*I have not been going through the counseling. But now, I realized much about counseling. And I have also realized that uterine evacuation goes hand in hand with counseling and then family planning. This one I did not know much about it… It is very important that you make her aware of the different types of family planning and the way we will do this uterine evacuation, being medical, being manual vacuum aspiration. So, that has really helped so much [to understand] that the woman, herself, will be able to decide what she wants, which choice she wants…This training really has helped change our attitudes because some of us used to think it should only be done to people who have been raped: they just sympathize with them, and induced abortion should not be done to others… –* Participant from Uganda

#### Training methodology

Participants highly appreciated the balance between theory and practice through role-plays and skill rehearsal. The humanistic anatomical models were critical for skills demonstration by facilitators and for hands-on practice by participants.

*The practice on the anatomic models and the exercises helped me assimilate the contents. However, we did not practice on [real] clinical cases, and the course was taught in a hurry.* – Participant from the DRC

Many participants agreed with the perceived short duration of the workshop and the lack of clinical practice reported in the previous quote. Participants suggested adding one to two more days to their workshop, including the opportunity to practice with real patients in clinical settings. In all three countries, there were no patients available for the day planned for practice at the clinic. In this regard, trainees suggested the following actions for the organization of future workshops:

*Prior to the training, we can liaise with facilities around to pool of patients possible for clinical practicum. Each case will offer an opportunity for further discussions. A day or two will need to be added for this purpose. A visit to one or two camps will help facilitators describe in clearer terms how services should be organized. –* Participant from Nigeria

Participants generally appreciated the quality of the training materials but also reported a few gaps. As reflected earlier on the data incompleteness of the competency checklists, participants reported the need for clear instructions on how to use these checklists to practice skills and validate competencies. In addition, due to local delays in organizing the workshop, several participants in the DRC regretted not receiving hand-out documents.

*No distribution of teaching aid before, during, or after the training. We will not know how to review the contents after the workshop*. – Participant from the DRC

*The materials are easy for the participants to understand. However, there is a need to improve on the instructions for the practical simulation and how co-participants can score themselves. –* Participant from Nigeria

**Fig. 2:**
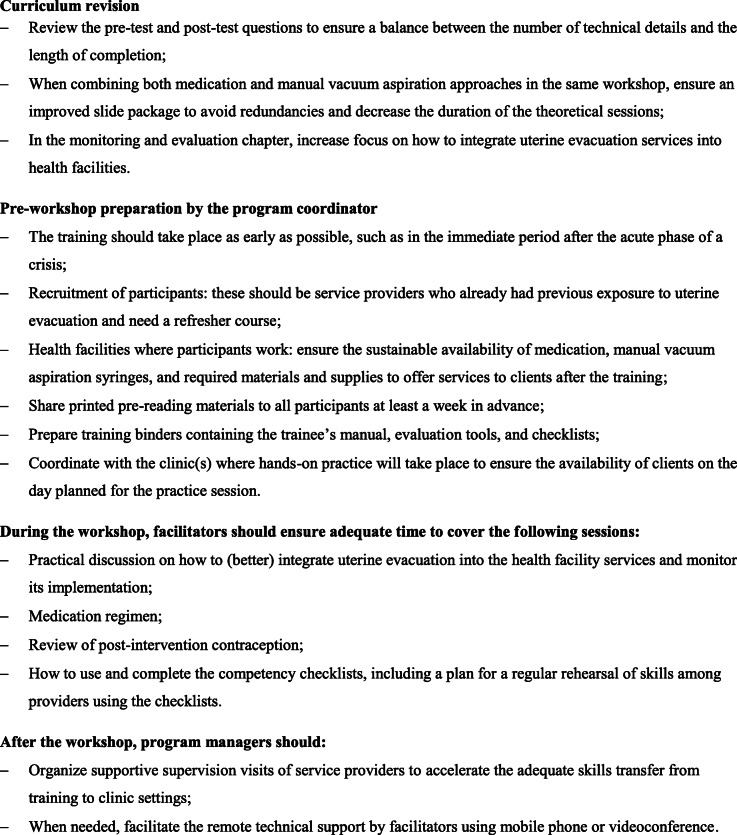
Summary of recommendations to improve the capacity development continuum

Participants shared other recommendations on how to improve their training experience. Figure 2 summarizes the key recommendations under four categories: curriculum revision, pre-workshop preparation, during the workshop, and after the workshop.

## Discussion

The newly developed S-CORT curriculum on uterine evacuation integrating manual vacuum aspiration and medication techniques was implemented in fragile or humanitarian contexts in Uganda, Nigeria, and the DRC. Results from the three workshops converged to suggest that the module contributed to increasing participants’ theoretical knowledge and possibly their technical and counseling skills. Equally noteworthy were their confidence building and positive attitudinal changes promoting a rights-based, fearless, non-judgmental, and non-discriminatory approach toward clients. Participants valued the hands-on, humanistic, and competency-based training methodology, although most regretted the short training duration and lack of practice on real clients.

We can draw several lessons to improve this S-CORT curriculum and the overall model as well as inform the design and implementation of new training curricula aimed at refreshing the knowledge and skills of service providers working in humanitarian settings.

First, this triple evaluation aligns with previous research on the S-CORT model, which suggests that the approach is respectful of human rights and quality of care principles in addition to being potentially effective in enhancing the knowledge and skills of existing trained service providers, strengthening their capacity, and changing their attitudes [15].

Combining medication uterine evacuation with manual vacuum aspiration within the same curriculum appeared feasible and indeed complementary as misoprostol and mifepristone are increasingly available in countries affected by fragility or crises [32, 33]. Additionally, universal access to such medications, which are part of the WHO Model List of Essential Medicines [34], adhere to contemporary standards on sexual and reproductive health and rights [35].

Second, it is important to remember the S-CORT capacity development strategy: a rapid on-the-ground training during the acute or post-acute phase of a crisis to refresh the knowledge and skills of service providers on a specific lifesaving intervention, which they learned in the past. However, all three workshops, although implemented in fragile or humanitarian contexts, did not occur in acute crisis settings, and many of the participants did not have former training on uterine evacuation. Therefore, humanitarian coordinators should be considerate of the operational context, available resources, and profiles and needs of participants when planning for an extension beyond the two-day core training. For instance, adding an extra day for values clarification and attitude transformation is a best practice in uterine evacuation programming and should be a prerequisite if not done previously. However, if resources for an extra training day are constrained, the S-CORT curriculum already covers the topic to some extent in a condensed session. For those without prior exposure to uterine evacuation, and particularly hands-on MVA skills, a more comprehensive workshop over five or more days with ample opportunities for clinical practice would better meet their training needs. For the training to be both effective and efficient, participants should be screened in advance and training materials adapted to ensure the course is the most appropriate to meet their learning needs, background, and professional objectives [36].

Third, evaluating programs in unstable and resource-limited settings raises the question of balancing feasibility with validity [37]. The evaluation of our pilots had the merit of adopting a multi-pronged approach to shed light on changes in knowledge (pre-test and post-test), participants’ and trainers’ experience and perspectives (qualitative methods), and the strengthening of competencies (competency checklists). Our experience speaks against using competency checklists as a training program evaluation tool but illustrates the usefulness and feasibility of a mixed-method approach using qualitative research in addition to pre-tests and post-tests. These interviews provided nuances to the results of the written tests by exploring important skill retention factors, such as attitudinal changes or confidence [38]. Competency-based training requires a checklist to allow an observer (ideally a co-trainer, and, if not feasible, a co-trainee) to systematically record the status and progress for each step of the clinical competency to be acquired by a trainee. Such checklists are part of the S-CORT competency-building approaches and were used in all three pilots. We initially planned to capitalize on the availability of these checklists and integrate them into the mixed-method documentation of the pilots. There were 63 steps for medication uterine evacuation and 78 for manual vacuum aspiration. Collecting and cleaning this vast amount of data for each participant was a daunting task, which we underestimated. The checklist forms collected from all three workshops showed that they were used but with considerable incompleteness and inconsistencies, which did not allow us to exploit the data. Therefore, competency-based checklists should be used as a support to build trainees’ competencies rather than a workshop reporting tool. In this respect, it is critical for facilitators to clearly explain how to use the checklist and verify that trainees do so correctly and systematically to evaluate one another reliably. Such a checklist has the added value of serving as an ongoing training job aid for providers to rehearse and boost their clinical skills periodically after the training [39].

Regarding knowledge testing, the increase of the average post-test score across countries and the rise by 25 percentage points among a few participants suggested that the curriculum could be overall effective in enhancing knowledge. In Uganda, the average pre-test score of 84% with a modest post-test increase of 5 percentage points may be due to the overall high level of knowledge of a relatively homogeneous group of service providers. In contrast, the Nigerian participants scored on average lower and had a modest post-test increase. With a third of participants having a lower post-test score, the overall performance in Nigeria could have been affected by the inadequate mix of community health extension workers, who do not provide uterine evacuation services, with other providers as well as post-workshop fatigue and reporting error considering the perceived complex and “bulky” set of questions. However, the fact that around half of the participants were community health extension workers (and one radiologist!) likely biased the results: they did not constitute the appropriate audience, which likely reduced their training self-efficacy and knowledge and skills retention [40]. Community health workers can play a critical role in preventing unsafe abortion and could have benefited from a curriculum that ensured better training utility and skills transfer. Such a curriculum could include, for instance, essential information for community awareness and mobilization, values clarification and attitude transformation, and even eligibility assessment for early medical abortion using a standardized checklist as demonstrated by the WHO [41]. The mismatch between participants and curriculum underscored the importance of offering the appropriate training to the right audience especially in resource-limited humanitarian settings.

Fourth, the recommendations summarized in Fig. 1 were valuable in improving the training module before its finalization. Although some of the recommendations may appear ordinary, especially for development settings, organizing and running capacity building events in humanitarian settings often face constraints in terms of security, time, material, and human resources. Immediate and longer-term transfer of learning may be influenced by a core set of factors, no matter the context [42]. Some of these factors emerged positively from the evaluation (acquisition of knowledge and skills, perceived relevance, attitudinal change, motivation, and confidence). Others, such as the in-clinic availability of supplies, materials, or job aids, should be improved to facilitate trainees’ autonomy to create opportunities to use their skills in health facilities.

Finally, the S-CORT approach relies on master trainers to travel to humanitarian settings. Traveling to the field, where trainees work, is a requirement but a significant limitation of the model, especially when movement restrictions are due to insecurity or infection control measures—the COVID-19 pandemic is an illustration of the latter [43]. In consequence, our model should adapt and integrate different training options that favor self-learning and remote teaching and mentoring through a blended approach. However, these mobile strategies rely on information technology and electronic platforms that may not be widely accessible to service providers working in humanitarian settings and would require further research. While uterine evacuation using medication may be suitable for mobile learning, manual vacuum aspiration requires hands-on coaching. Mobile learning applications or modules should not suffer from a reductionist view that only promotes a mobile platform and neglects the complex relationship between adult learning principles and technology [44]. Therefore, the development of future mobile learning strategies should also be underpinned by proven learning approaches, including collaboration, reflection, building on prior experiences, and focusing on improving practice instead of evaluation [45].

## Conclusions

Uterine evacuation is a lifesaving intervention, and access to these services has been a significant gap in humanitarian settings. When the lack of skilled human resources is a barrier to services, the S-CORT strategy could offer a rapid hands-on refresher training opportunity for service providers needing an update, and, therefore, contribute to achieving the implementation of the MISP. Such a capacity-building approach could be useful in humanitarian and fragile settings as well as in development settings with limited resources, however, only within an overall effort to strengthen other building blocks of the health system—a system that meets the SRH needs and rights of women, girls, and the whole community.

## Data Availability

Data is available upon reasonable request from the corresponding author.

## Abbreviations

DRC: Democratic Republic of Congo
MISP: Minimal Initial Service Package for Sexual and Reproductive Health in Humanitarian Settings
MVA: Manual Vacuum Aspiration
S-CORT: Sexual and Reproductive Health Clinical Outreach Refresh Training
SRH: Sexual and Reproductive Health
VCAT: Values Clarification and Attitude Transformation
WHO: World Health Organization
